# The Riddle of Pain

**Published:** 1896-12-05

**Authors:** 


The Hospital

A Journal of

The Medical Sciences and Hospital Administration, with

AN EXTRA NURSING SUPPLEMENT.

Vol. XXI.—No. 532.] [Dec. 5, 1896.

### The Riddle of Pain.

At the Boston celebration of the jubilee of anæsthesia the other day Dr. S. Weir Mitchell, so well known to the profession and the public in connection with treatment by massage, read an original poem on the "Birth and Death of Pain." The mystery of pain is as profound as that of creation. The purpose of pain, the objective of so many enquirers in all ages, is pronounced by Dr. Mitchell to be a "riddle," and a riddle to which "earth can give no answer." The English surgeon, James Hinton, whose too premature death inflicted an irreparable loss upon philosophic medical thought, and who himself was the victim of almost unceasing pain during the greater part of his mature life, differed widely from Dr. Mitchell in the reading of this "riddle of the painful earth." He thought that pain was the minister of a very lofty purpose, and that he himself could see quite clearly what that purpose was.

In pronouncing the judgment that the "purpose of pain is a riddle to which earth has no answer," Dr. Weir Mitchell, we are inclined to think, has forgotten to take counsel of his "Bacon." If he had really looked to earth, in Bacon's way, he might not indeed have found what he himself would have called "the answer" to the riddle; but that he would have found an answer, or two, or several, we are quite convinced; and the putting of all the answers together might have been considered an almost perfect solution of the mystery.

"Let us consider the actual facts of the case," Bacon would have said. Here is a man with a painful broken arm. Does the pain serve any special purpose, any purpose which might not as well have been served without it? Most assuredly it does. But for the pain, the average man—not, perhaps, the scientific man, but the average man—would not pay heed to his injury; would not, in fact, devote the necessary time and trouble to its perfect repair. We have to consider what the average man is for the purposes of this discussion. He is not the average modern American, or modern European, tinctured with all the culture, all the science, all the high morality of the modern world. He is the typical person of no education who has made up the generations of men from the earliest times when mankind emerged from the practically brute condition into dawning moral consciousness. That is the average man to be considered when we ask what may be the purpose of pain. Has pain had a purpose of any kind for all those countless generations of the uncultured past who have constituted the solid mass of mankind? Most assuredly it has had a purpose—many purposes. It has compelled attention to injured structures; it has enforced rest and sleep by the distress of weariness; the taking of food by the tortures of hunger; and, in short, has been the general indicator and corrector for man and beast in the exercise of physical and physiological energy of every kind. Not only so, but the moralist and the religious teacher will unite in insisting that the educational value of pain in the regions of morals and religion has been, and continues to be, incalculable. So far from agreeing with Dr. Weir Mitchell that pain has no purpose in the world, we affirm that one of the most obvious of all the facts connected with pain is its definite and incalculable value, as an indicator, a corrector, an educational force, alike in the physical, the mental, and the moral spheres. Its purpose, indeed, is as plain to the Baconian reasoner as that of the moon in the sky.

If these things be so, is it either right or philosophical to interfere with the action of pain? The question is surely as childish as could well be asked. That which is essential for one period of human development may not be essential for another. The sharp physical stimuli, the clubs and spears of the early savage, are not needed by the later races of men. In earlier times hunger, thirst, fear of wounds from enemies, the most elementary of all sensations were needed to compel, even the highest races of men, to do the best that was in them. In our times there are millions who work in obedience to motives altogether different from the driving forces of hunger, cold, and physical fear. Ambition compels exertion, duty, mere love of work. And so the element of painfulness, being less and less needed, plays a less and less conspicuous part as a driving and correcting force in the world. Will pain, or the possibility of pain, ever be eliminated from the experience of man, or "killed," as Dr. Weir Mitchell might prefer to put it? Most probably not, so long as man is endowed with his present nervous system. But it is possible, nay, it is quite easy, to imagine a time when mankind in general shall have reached such a stage of mental capacity and culture, such a wide and masterful victory over nature, such a degree of physical vigour and material prosperity, that pain shall be a very exceptional fact in his experience. This is the goal at which a philosophical medical science must at any rate aim, with all the energy of which it is capable; and, among other purposes of pain which are obvious to the seeing man, is this very one of compelling unceasing and high mental effort for its own absolute and permanent extinction as a factor in human affairs. If it be asked at what period in the future this Utopia of painlessness is likely to be reached, we would refer the questioner for an answer to the prophetic medicine of America.

